# Urinary iodine excretion and optimal time point for sampling when estimating 24-h urinary iodine

**DOI:** 10.1017/S0007114523000326

**Published:** 2023-10-28

**Authors:** Janna Eriksson, Lars Barregard, Gerd Sallsten, Balazs Berlinger, Stephan Weinbruch, Sofia Manousou, Dag G. Ellingsen, Helena Filipsson Nyström

**Affiliations:** 1 Department of Internal Medicine and Clinical Nutrition, Institute of Medicine, Sahlgrenska Academy, University of Gothenburg, Gothenburg, Sweden; 2 Praktikertjänst AB, Skövde, 54130, Sweden; 3 Occupational and Environmental Medicine, Department of Public Health and Community Medicine, Institute of Medicine, Sahlgrenska Academy, University of Gothenburg, Sahlgrenska University Hospital, Gothenburg, Sweden; 4 National Institute of Occupational Health, Oslo, Norway; 5 Department of Animal Hygiene, Herd Health and Mobile Clinic, University of Veterinary Medicine, Budapest, Hungary; 6 Institute of Applied Geosciences, Darmstadt Technical University, Darmstadt, Germany; 7 Frölunda Specialist Hospital, Västra Frölunda, Sweden; 8 Department of Endocrinology, Institute of Medicine, Sahlgrenska University Hospital, Gothenburg, Sweden; 9 Wallenberg’s Centre for Molecular and Translational Medicine, Sahlgrenska University Hospital, Gothenburg, Sweden

**Keywords:** Iodine, Urinary iodine concentration, 24-h urinary iodine excretion, Estimated 24-h urinary iodine excretion, Urine sampling, Sampling time, Variability

## Abstract

Iodine deficiency may cause thyroid dysfunction. The iodine intake in a population is measured by urinary iodine concentration (UIC) in spot samples or 24-h urinary iodine excretion (24UIE). 24UIE is considered the gold standard and may be estimated using an equation including UIC, urinary creatinine concentration, sex and age (e24UIE). The aims of this study were to evaluate the preferable timing of UIC when using this equation and assess the variability of UIE. Sixty healthy non-smoking women (*n* 31) and men (*n* 29) were included in Gothenburg, Sweden. Twelve urine samples were collected at six fixed times on two separate days. Variability was calculated for UIC, 24UIE, e24UIE, iodine excretion per hour (iHr) and UIC adjusted for creatinine and specific gravity. Median 24UIE was 156 µg/24 h and the median UIC (all spot samples) was 104 µg/l. UIC (*P* < 0·001), 24UIE (*P* = 0·001) and e24UIE (*P* < 0·001) were significantly higher in men. e24UIE was relatively similar to 24UIE. However, when e24UIE was calculated from UIC in the first void, it was about 15 % lower than 24UIE (*P* < 0·001). iHr was lowest in the morning and highest in the afternoon. Median iHr was higher in men (7·4 *v*. 5·3 µg/h, *P* < 0·001). The variability of UIE was higher within individuals than between individuals. This study suggests that most time points for estimation of individual 24UIE are appropriate, but they should preferably not be collected in the first void.

Iodine is an important nutrient for human health and normal thyroidal function as it is an essential trace element in thyroid hormones. Iodine deficiency is therefore a common cause of hypothyroidism and goitre globally^(1)^. In 1993, WHO and UNICEF recommended universal salt iodisation as the most optimal strategy to ensure iodine sufficiency for all individuals worldwide^(2)^. The challenge of preventing and curing iodine deficiency is ongoing and positive results have been achieved^(3)^; however, regular monitoring of iodine intake in various populations is important for maintaining iodine sufficiency^(4)^.

Monitoring of iodine nutrition requires a valid biomarker. As goitre is a somewhat late indicator of iodine deficiency^(5)^, measurement of thyroid volume is only used in special cases. The dominant and recommended method for examining iodine intake at a population level is to measure iodine excretion from median urinary iodine concentration (UIC) in spot samples. According to the definitions set by WHO for school-age children at a group level, a median UIC 50–99 µg/l corresponds to mild deficiency, 20–49 µg/l to moderate deficiency and < 20 µg/l to severe iodine deficiency, while a median UIC 100–199 µg/l corresponds with adequate iodine nutrition^(2)^. This method has, however, some challenges^(2)^. These UIC levels cannot be extrapolated to adults directly, as adults have larger urine volumes; it has been suggested that an adult-specific cut-off range of 60–70 µg/l can be used for iodine sufficiency^(3)^.

In addition, spot UIC shows a diurnal rhythm. Over 24 h, approximately 90 % of an individual’s iodine intake is excreted^(2,5,6)^. Therefore, 24-h urinary iodine excretion (24UIE) is a better biomarker for iodine nutrition than spot UIC, where fourteen repeated samples may be needed to estimate an individual’s iodine intake^(7,8)^. Spot UIC has a high day-to-day variability^(9)^ and is also dependent on seasonal variations in iodine intake^(9–11)^, differences in fluid intake^(7)^ and the diurnal rhythm of UIE^(12,13)^. Hence, the timing of spot sample collection is of importance, which is confirmed in some^(9,12,14)^ but not all studies^(15)^. 24UIE is currently the gold standard^(2,8)^, but the challenge of obtaining complete 24-h urine collection limits its use.

If it is not possible to measure 24UIE, estimated 24UIE (e24UIE) can be calculated by an equation based on spot UIC, spot urinary creatinine concentration and standard population data for the expected 24-h creatinine excretion based on sex and age^(16,17)^. Sometimes, UIC concentration adjusted for diuresis by urinary creatinine is used (UIC/Cr).

A systematic review from 2015 underlines the importance of further studies to investigate whether the time of sampling is of importance when estimating 24UIE: this is a knowledge gap^(18)^. We therefore aimed at determining the optimal time point to estimate 24UIE. We also explored, for the first time, the excretion rate of iodine per hour over 24 h.

## Methods

### Participants and background information

A description of the study population and sample collection has been published elsewhere^(19)^. In total, eighty-two individuals were asked to join the study, eleven refused and eleven did not fulfil the study entry criteria. Finally, sixty healthy participants (thirty-one women and twenty-nine men) without medical history of diabetes, kidney disease, hypertension or self-reported medications for chronic disease were included and examined in 2012–2013. All subjects were non-smoking and were mainly employees or students at the University of Gothenburg and Sahlgrenska University Hospital in Gothenburg. The age range was 21–64 (mean 34) years. Most participants were born in Sweden, but twenty-two subjects were born in other countries (Iran, Iraq, USA, Russia, Finland, South Korea, Kyrgyzstan and Ukraine). They all completed a short questionnaire about relevant food habits and personal information such as country of birth, height and weight^(19)^. Median BMI was 23 kg/m^2^ (range 19–44), and median fish consumption was 2 meals/week (range 0–7). One female subject was the only vegetarian. The participants had no dietary restrictions during the study.

### Urine sampling and study design

Complete 24-h urine collection was conducted at specified times of the day on two different days separated by an interval of approximately 1 week. Sampling was performed during weekdays. In the morning of the starting day, upon rising, participants were instructed to discharge the first void of the day, recording the date and time as the starting point for the 24-h urine sampling. The participants were instructed to urinate at six fixed times (09.30, 12.00, 14.30, 17.30, 22:00 hours and first void the next morning). They were instructed to collect each void at the specific time in a separate bottle, recording the time of each void. If they needed to urinate in between the fixed times, they used the next bottle and then filled it again at the specified time point. On the next morning, they collected the first void of the day, representing the overnight urine sample. Participants were instructed to store their urine samples in a refrigerator and return all samples on the day they completed the collection.

Spot urine samples were collected in 1000 ml high-density polyethylene bottles with polypropylene screw caps (Bibby Sterilin Ltd) using no preservatives. Total volume and collection times were recorded for each urine sample. All urine samples were aliquoted into 2 ml polypropylene micro tubes (Sarstedt) and frozen within 8 h (–80°C).

### Chemical analyses

Analysis of UIC was performed at the National Institute of Occupational Health in Oslo, Norway. The laboratory regularly takes part in external quality control programs. Methods for metal analyses in urine, as well as levels and variability of twenty-two elements in 24-h urine, have been described in detail elsewhere^(20)^. Urine samples were analysed by inductively coupled plasma sector-field MS. Seronorm^TM^ Trace Elements (Seronorm^TM^, Sero AS) urine quality control materials were used for quality assurance. Two quality control samples (Seronorm L1 (lot 1403080) and Seronorm L2 (lot 1403081)) were used for urine: the respective UIC results (mean) compared with recommended values were 88 (sd 9·6) µg/l (*n* 16) *v*. 105 µg/l and 273 µg/l (sd 9·8) (*n* 16) *v*. 297 µg/l.

A UIC subsample (*n* 22) was analysed at the Department of Clinical Nutrition, University of Gothenburg (Gothenburg, Sweden) using the Pino modification of the Sandell–Kolthoff reaction^(21)^. The laboratory is evaluated for analytical accuracy every 3 months and successfully participates in the EQUIP network (US Centers for Disease Control and Prevention). Validation of the UIC analysis against inductively coupled plasma MS at the Genomics and Biomarkers Unit in Finland is performed as part of the EUthyroid project (Helsinki, Finland). The two methods had a high Spearman’s coefficient of correlation (*r*_*s*_ = 0·96, *P* < 0·001).

Analyses of creatinine in urine were performed in fresh urine, kept at 4°C until analysis within 3 d of collection, using Roche Creatinine enzymatic assay on a Cobas 6000 analyser (Roche Diagnostics Scandinavia AB, Sweden) with a limit of detection of 0·01 g/l and traceable to international standardisation with isotope dilution MS.

### Calculated variables

- UIC **(**µg/l). UIC is the iodine concentration in spot urine samples.
- UIC/Cr (µg/g creatinine). UIC from spot urine sample divided by urinary creatinine concentration.
- e24UIE (µg/24 h). Estimated 24UIE formula: iodine (µg/l)/((urinary creatinine (g/l)/expected creatinine excretion (g/d))^(17)^.
- 24UIE (µg/24 h). Iodine (µg) during 24-h collection (summed for each spot sample) and adjusted to 24 h.
- Iodine excretion per hour (iHr) (µg/h). Iodine in µg (UIC × volume) divided by the collection time in hours.

Since the distributions were skewed, descriptive results are reported as geometric means (sd). Normality was tested with the Shapiro–Wilk test and visually assessed by Q-Q plots and box plots. Outliers were visualised by scatter plots, and one outlier was then excluded from the analysis (apart from the intra-class correlation (ICC), see below). Median and mean values for total urine volume, 24-h urinary creatinine, UIC, 24UIE and e24UIE were calculated from individual 2-d means (except from one subject who only collected urine on 1 d). Spearman’s correlation coefficient (*r*_*s*_) was used to compare e24UIE at various points of time with 24UIE in the total group as well as separately in men and women. The Mann–Whitney independent sample test was used when comparing differences in iodine intake (e24UIE, UIC and 24UIE) and comparing iHr between men and women.

For each of the six sampling time points, the difference and the ratio between e24UIE and 24UIE were calculated for all participants and for men and women separately. Wilcoxon’s signed rank test was used to assess whether the differences were significantly different from zero.

The within- and between-individual variance components were estimated after natural log-transformation of 24UIE and e24UIE for the six different time points using PROC MIXED in the SAS software package (version 9.4 SAS Institute). The estimated ratio of the between-individual biomarker variance to total observed variance (the ICC) was calculated. The attenuation in a hypothetical log(exposure) to log(response) relationship in an individual-based study design was calculated, that is, the ratio between the regression slope estimated in the study (*β*_*est*_) and the true regression slope (*β*_*true*_). The degree of attenuation for a given measure of 24-h iodine was determined from the estimated variance components using the relationship:

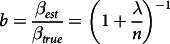

where bias is 1–*b*, *λ* = *σ*^*2*^_*wY*_/*σ*^*2*^_*bY*_, *n* is the number of repeated measurements per individual, *σ*^*2*^_*wY*_ is the within-individual variance and *σ*^*2*^_*bY*_ is the between-individual variance^(22)^. CV was determined from: CV = √(*e^*(*σ*^*2*^_*wY*_)–1)^(23)^.

Data were processed and analysed in Excel 2016 (Microsoft) and SPSS version 24.0 (IBM). Statistical significance was set at *P* < 0·05. iHr and iodine excretion in 24-h sampling were calculated from urinary concentrations, volumes and sampling times. For the assessment of variability, the UIC from the two separate samples at each time point over 24 h had to be used.

## Results

All participants provided urine samples with a total 24-h volume > 800 ml and total sampling time > 20 h except for one participant who missed part of the overnight sample on one of the two urine collection days. Sampling was reported to be complete for all other participants. Total urine volume and analyses of 24-h creatinine confirm a good degree of completeness for urine collections^(19)^. One subject collected all urine from 14.30 hours at 22.00 hours instead of urinating at the fixed time of 17.30 hours; however, the 24-h sampling for this person was still complete and was therefore included in analyses.

One outlier (24UIE 3463 µg) was found when analysing urine samples from day 2 and was therefore excluded from the subsequent statistical analyses, apart from the ICC. We have been in contact with this individual and have not found any reason why the iodine level over day 2 was exceptionally high.

### Urinary iodine among participants

The urinary iodine levels (UIC, 24UIE and e24UIE) of all subjects, and men and women separately, are described in Table 1. Median urine volume over 24 h was 1·58 l for all subjects, 1·53 l for men and 1·61 l for women. Median 24-h creatinine concentration was 0·94 g/l for all subjects, 1·26 g/l for men and 0·76 g/l for women. Total urinary volume and creatinine excretion have also been described in more detail elsewhere^(19)^. Median UIC was 104 µg/l for all subjects, 119 µg/l for men and 83 µg/l for women. Median 24UIE was 156 µg/24 h for all subjects, 174 µg/24 h for men and 130 µg/24 h for women. Median e24UIE was 141 µg/24 h for all subjects, 156 µg/24 h for men and 126 µg/24 h for women. There were significant differences in iodine excretion between men and women when comparing median UIC (*P* < 0·001), median 24UIE (*P* = 0·001) and median e24UIE (*P* < 0·001).

**Table 1.** Iodine status of the study subjects (calculated from individual 2-d means)

Parameter	All subjects (*n* 60)*				Men (*n* 29)*				Women (*n* 31)			
	Mean	Range	Median	IQR	Mean	Range	Median	IQR	Mean	Range	Median	IQR
UIC (μg/l)	120	9–576	104	60–162	136	12–576	119	74–177	106	9–444	83	50–141
24UIE (μg/24 h)	166	53–461	156	110–200	191	61–461	174	135–244	144	53–296	130	100–183
e24UIE (μg/24 h)	161	21–642	141	101–193	177	32–642	156	112–211	146	21–583	126	93–181

24UIE, 24-h urinary iodine excretion; e24UIE, estimated 24-h urinary iodine excretion; IQR, interquartile range; UIC, urinary iodine concentration.

* Day 2 outlier data for one subject excluded.

### Diurnal variation and the urinary excretion per hour over a 24-h period

**Fig. 1.**
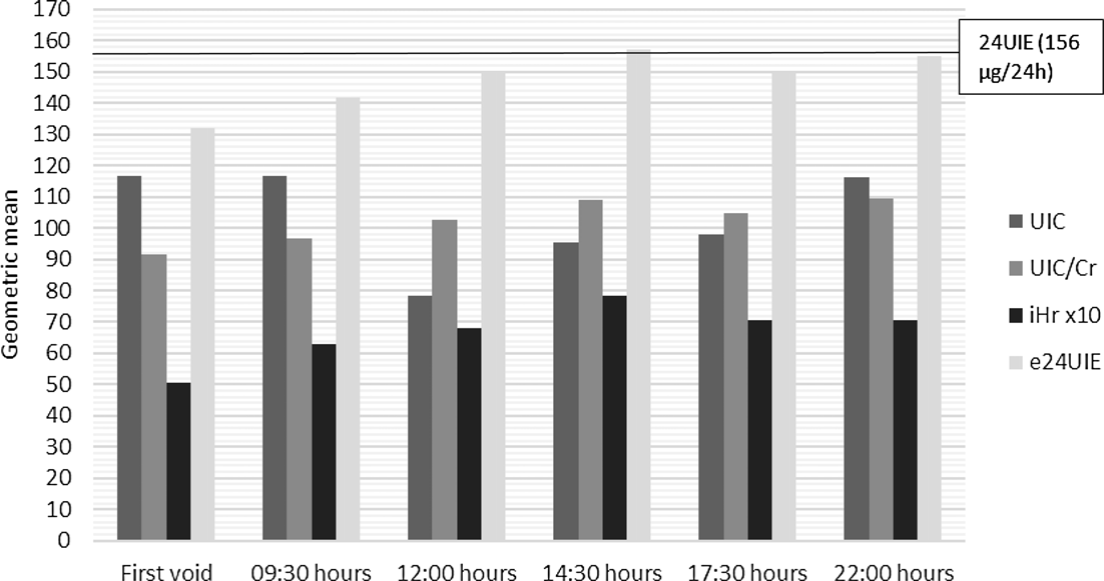
Geometric mean for iodine concentration spot urine samples (UIC as µg/l), UIC adjusted for urinary creatinine (UIC/Cr as µg/g creatinine), iodine excretion per hour (iHr as µg/h) and estimated 24-h urinary iodine excretion (e24UIE as µg/24 h). The geometric mean of 24-h urinary iodine excretion (24UIE as µg/24 h) is represented by a line for comparison. Day 2 outlier data for one subject excluded. The geometric mean is calculated from individual 2-d means.

Fig. 1 shows that the peak iHr occurred in the afternoon (14:30 h) and was lowest in the first morning urine (first void). UIC/Cr follows the pattern of iHr relatively well, while this is not the case for UIC which peaked in the morning and at night with lower levels in the middle of the day. Median iHr was higher for men than women (7·4 and 5·3 µg/h, *P* < 0·001).

### Validity of estimated 24-h urinary iodine excretion: the gold standard

**Fig. 2.**
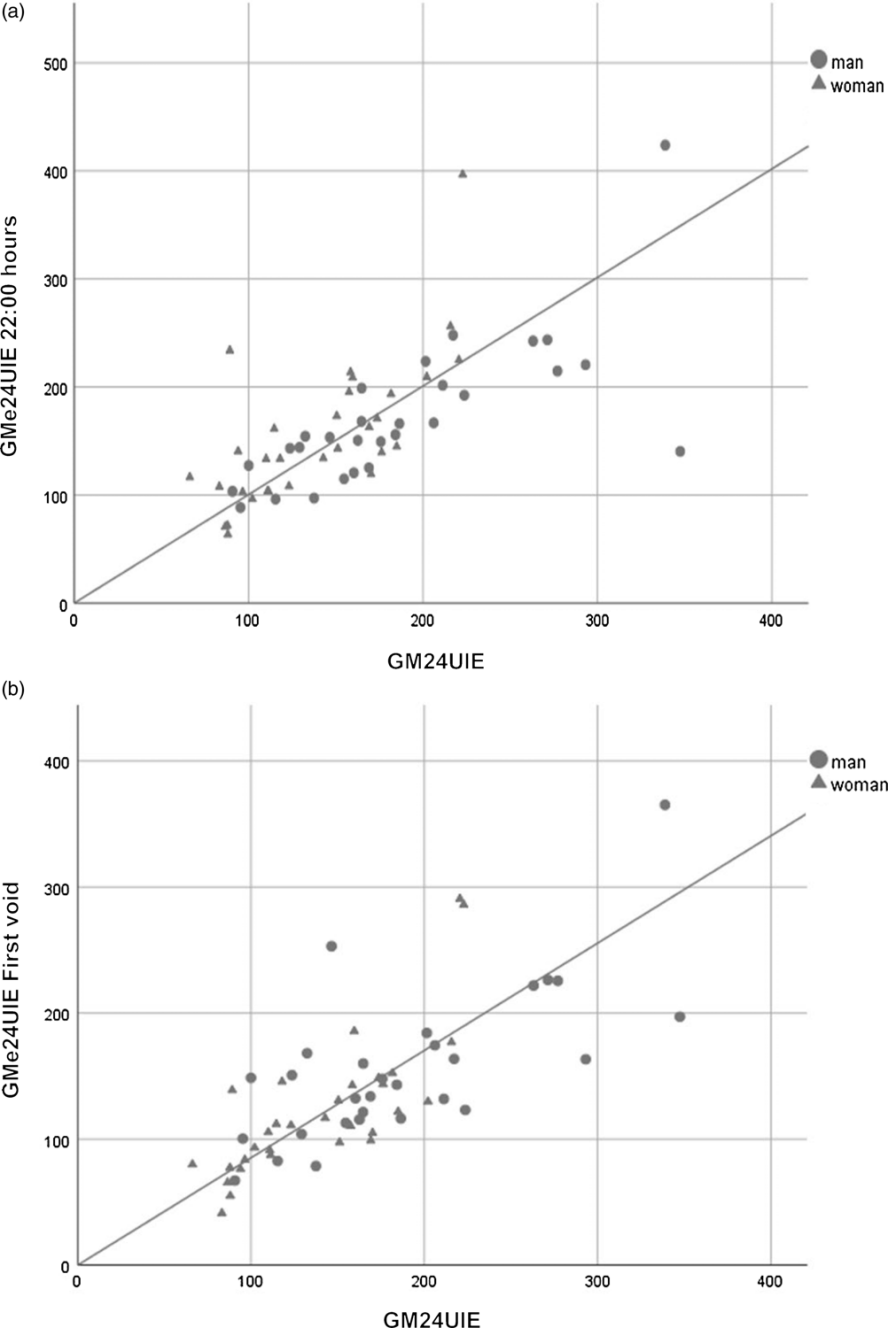
Scatter plots showing positive correlation between (a) geometric mean of iodine excretion over 24-h sampling (GM24UIE) and geometric mean of estimated 24-h excretion based on an evening sample (GMe24UIE 22.00 hours) (*r*_*s*_ = 0·743) and (b) gm24uie and geometric mean of estimated 24-h excretion based on a first void sample (GMe24UIE first void) (*r*_*s*_ = 0·745). The regression lines display the association between the two variables and without an intercept. Day 2 outlier data for one subject excluded. The geometric mean is calculated from individual 2-d means.

The four different ways of measuring urinary iodine (UIC, UIC/Cr, e24UIE and iHr) in comparison with the geometric mean 24UIE (156 µg/24 h) are shown in Fig. 1. Geometric mean values and standard deviations (range) are also presented in Table 2. Table 3 shows the mean of the ratio between 24UIE and e24UIE for all time points. The difference (24UIE subtracted by e24UIE) was calculated and tested if significantly different from zero. When comparing the geometric mean of e24UIE and 24UIE, respectively, the optimal time points seem to be at 14.30 and 22.00 hours (Fig. 1), but all voids from 09.30 hours onwards showed good agreement (Table 3). The most inappropriate time point would appear to be the first void in the morning (Fig. 1), which differs significantly from 24UIE (Table 3). When analysing the most optimal time points, correlation was highly significant at 14.30 hours (*r*_*s*_ = 0·71, *P* < 0·001) and 22.00 hours (*r*_*s*_ = 0·74, *P* < 0·001). When comparing the geometric means of 24UIE and e24UIE for men and women separately, the correlations were high at 14.30 and 22.00 hours (*r*_*s*_ > 0·7, *P* < 0·001) (Fig. 2(a)). In addition, correlation was high for the first void (*r*_*s*_ = 0·75, *P* < 0·001) (Fig. 2(b)), even though e24UIE was systematically too low.

**Table 2.** Iodine excretion parameters at different sampling time points and 24UIE (calculated from individual 2-d means) as reference for the sixty participants (excluding day 2 outlier data for one subject)

Time/parameter	Geometric mean	GSD	Range	Median
First void				
UIC (µg/l)	117	1·6	37–368	117
UIC/Cr (µg/g)	92	1·5	39–238	89
iHr (µg/h)	5	1·6	1–11	5
e24UIE (µg/24 h)	132	1·5	52–369	129
09.30 hours				
UIC (µg/l)	117	2·0	22–406	126
UIC/Cr(µg/g)	97	1·6	29–261	90
iHr (µg/h)	6	1·7	2–18	6
e24UIE (µg/24 h)	142	1·6	51–441	129
12.00 hours				
UIC (µg/l)	78	2·0	15–290	71
UIC/Cr(µg/g)	103	1·5	42–306	95
iHr (µg/h)	7	1·6	2–17	7
e24UIE (µg/24 h)	150	1·5	74–477	145
14.30 hours				
UIC (µg/l)	95	1·7	28–261	92
UIC/Cr(µg/g)	109	1·5	47–318	99
iHr (µg/h)	8	1·6	3–27	7
e24UIE (µg/24 h)	157	1·5	75–553	151
17.30 hours				
UIC (µg/l)	98	1·8	24–254	94
UIC/Cr (µg/g)	105	1·5	44–296	100
iHr (µg/h)	7	1·6	3–21	7
e24UIE (µg/24 h)	149	1·5	65–406	147
22.00 hours				
UIC (µg/l)	116	1·7	34–261	111
UIC/Cr (µg/g)	109	1·5	51–323	104
iHr (µg/h)	7	1·6	3–19	7
e24UIE (µg/24 h)	155	1·5	64–427	157
Over 24 h				
24UIE (µg/24 h)	156	1·7	67–361	156

24UIE, 24-h urinary iodine excretion; e24UIE, estimated 24-h urinary iodine excretion; GSD, geometric standard deviation; iHr, iodine excretion per hour; UIC, urinary iodine concentration; UIC/Cr, UIC divided by urinary creatinine concentration.

**Table 3.** Mean of the ratio between e24UIE and 24UIE at different sampling time points (calculated from individual 2-d means)

Time	All subjects (*n* 60)*		Men (*n* 29)*		Women (*n* 31)	
	Mean of ratio	*P*	Mean of ratio	*P*	Mean of ratio	*P*
First void	0·88	<0·001	0·88	0·003	0·88	0·011
09.30 hours	0·96	NS	0·92	NS	1·00	NS
12.00 hours	1·01	NS	0·93	NS	1·07	NS
14.30 hours	1·06	NS	1·01	NS	1·11	NS
17.30 hours	1·00	NS	0·95	NS	1·04	NS
22.00 hours	1·04	NS	0·96	NS	1·11	NS

24UIE, 24-h urinary iodine excretion; e24UIE, estimated 24-h urinary iodine excretion; NS, not statistically significant (*P* > 0·05).

* Day 2 outlier data for one subject excluded.

### Variability in 24-h excretion of iodine

The variability between days dominates for 24UIE and for the different time points for e24UIE (Table 4). The ICC for 24UIE was 0·35. The within-individual variance component was 0·178 (calculated from the total variance of 0·276 and the ICC of 0·354) which can also be expressed as CV of 44 % (34 % if excluding outlier sampling day 2). As expected, excluding one outlier reduced the total variance. In an epidemiological study examining outcome as function of iodine exposure using an individual-based design, the slope would be lower than the true slope, that is, the bias of the slope (*b*) would be large (47–90 %), if the iodine exposure is estimated from only one sample per individual. To achieve 70–80 % of an assumed true slope, at least five samples per individual are needed: either 24UIE or e24UIE during different days using one of the sampling time points 09.30, 12.00 and 22.00 hours or the first void.

**Table 4.** Total variance, ICC, *λ* and *b* for 24UIE and e24UIE at different sampling time points (using two separate samples at each time point over 24 h). Attenuation (*b*) in a hypothetical log (exposure) to log (response) relationship in an individual based study design is presented

Time/parameter	Total variance	ICC	*λ*	*b* (*n* 1)*	*b* (*n* 2)*	*b* (*n* 5)*
First void						
e24UIE	0·268	0·439	1·28	0·44	0·61	0·80
e24UIE (excl)†	0·233	0·454	1·21	0·45	0·62	0·81
09.30 hours						
e24UIE	0·404	0·407	1·46	0·41	0·58	0·77
e24UIE (excl)†	0·288	0·458	1·18	0·46	0·63	0·81
12.00 hours						
e24UIE	0·322	0·396	1·52	0·40	0·57	0·77
e24UIE (excl)†	0·225	0·532	0·88	0·53	0·69	0·85
14.30 hours						
e24UIE	0·358	0·104	8·63	0·10	0·19	0·37
e24UIE (excl)†	0·286	0·161	5·21	0·16	0·28	0·49
17.30 hours						
e24UIE	0·345	0·142	6·06	0·14	0·25	0·45
e24UIE (excl)†	0·273	0·162	5·18	0·16	0·28	0·49
22.00 hours						
e24UIE	0·270	0·299	2·25	0·31	0·47	0·69
e24UIE (excl)†	0·221	0·296	2·37	0·30	0·46	0·68
Over 24 h						
24UIE	0·276	0·354	1·82	0·35	0·52	0·73
24UIE (excl)†	0·195	0·428	1·34	0·43	0·60	0·79

24UIE, 24-h urinary iodine excretion; e24UIE, estimated 24-h urinary iodine excretion; ICC, intra-class correlation (ratio of the between-individual variance/total observed variance); *λ*, within-individual variance/between-individual variance.

* *b* (estimated slope/true slope) with *n* samples/individual.

† Day 2 outlier data for one subject excluded.

## Discussion

### Major findings

Our study included 720 urine samples from sixty healthy individuals. It compared different ways of evaluating UIC in relation 24-h iodine excretion to determine whether urinary iodine sampling can be improved. Our results showed that iHr is highest in the afternoon. The excretion rate reflected diurnal variation better than UIC. According to our results using e24UIE, UIC should be measured in urine samples after 09.30 hours and not in the first void.

### Urinary iodine compared with other national studies

Iodine intake in populations is nowadays mainly monitored by urine samples and measuring UIC^(2)^. However, due to its limitations, other methods are now being developed. Blood samples and measuring thyroglobulin as a marker for iodine deficiency have been studied and might be a way of monitoring iodine intake in the future due to lower variability^(24,25)^. For now, the most common way of monitoring iodine intake remains the collection of urinary samples: improvement of standard methods is therefore still relevant. It is important to perform this type of study under conditions of iodine sufficiency since iodine deficiency may affect the physiology of iodine excretion. We performed the present study in an adult population of men and women sampled in 2012 and 2013. UIC levels are presented in Table 1 and are in accordance with the cut-off levels suggested for iodine sufficiency in adults^(2)^. This also agrees with the results in several local studies^(26,27)^ and a national survey in 2007 in school-age children that confirmed iodine sufficiency in the Swedish population^(28)^. The most important sources for iodine intake in Sweden are iodinated salt, sea food and dairy products as animal feed contains iodine^(29)^.

### Estimated 24-hour urinary iodine excretion compared with 24-h urinary iodine excretion and the value of specific time points when measuring iodine in urine samples

24UIE is a more specific method than UIC to estimate and measure iodine excretion^(10)^ and is often used as a reference when validating other methods^(6)^. It is not surprising that e24UIE^(17)^ was the variable that was most comparable to the true 24UIE, since e24UIE considers creatinine, UIC and expected creatinine excretion. Creatinine is often used as a correction factor for the urine volume^(8)^ but has been questioned by WHO and considered ‘unreliable’ when correcting iodine concentration^(2)^. According to Knudsen and colleagues^(17)^, the algorithm for e24UIE provides a better and unbiased estimate of iodine excretion in epidemiological surveys than UIC/Cr. Using the first void sample for e24UIE, we underestimated the individual 24UIE by on average 12 %, while the differences between e24UIE and 24UIE were small and non-significant at the time points from 09.30 h and onwards (Table 3).

It is well known that UIC varies during the day. A Swiss study from year 2000 (*n* 3023 adult subjects) reported that iodine concentration follows a diurnal rhythm with a nadir at 11.00 hours and the highest value at 22.00 hours. It follows a pattern with successive increase during the day with peaks 4 h after every daily meal, indicating that the iodine excretion is relatively rapid after iodine intake^(12)^. Also, a Danish study suggested that certain time points during the day are more preferable than others when estimating 24UIC^(9)^, since the fasting morning sample seems to underestimate the true value. Our results agree with these studies and show that the first void sample underestimated daily iodine excretion and the optimal time point for sampling would appear to be later during the day.

However, if the most important aim of a study is not to estimate the true 24UIE but to examine the relation between e24UIE and a health outcome, then spot samples from late in the evening or in the morning until noon seem to be the best choice (Table 4). According to our results, at least five samples per individual are needed to achieve a bias of not more than 20–30 % compared with the true slope.

### Iodine excretion per hour

In our study, iHr has been calculated in humans for the first time. This is important since it reflects iodine excretion from the kidneys and gives new knowledge on basic human physiology. It is also clear that iHr varies during the day (Fig. 1), probably mainly depending on food habits. The correlation between urinary iodine and daily food intake is well known, and it is estimated that urinary iodine approximately reflects food intake over the previous few hours^(12)^. In Sweden, and in Gothenburg where the study took place, normal bedtime hours are between 22.00 and 06.00 hours during workdays. Work hours are normally between 08.00 and 17.00 hours, with a 1-h lunch break at 12.00–13.00 hours. It is therefore reasonable to assume that breakfast takes place between 06.00 and 07.00 hours, lunch between 12.00 and 13.00 hours and dinner between 18.00 and 21.00 hours. Fig. 1 shows the variability of iHr and that the excretion rate per hour was as highest at 14.30 hours, presumably reflecting iodine intake during lunch. The 24-h variation of urinary specific gravity does not follow the same pattern as iHr since it peaks in the morning^(19)^ and shows that iHr is likely not dependent on the concentration of solutes in urine. Median iHr was higher in men than in women. This is probably because of differences between men and women in iodine intake during the day, which is also reflected by differences in urinary iodine between men and women in our study population (Table 1). This agrees with a Norwegian population study which showed that self-estimated iodine intake and 24UIE were higher among men than women in a Nordic population^(30)^.

### UIC/Cr as a marker for iodine excretion

UIC is highest in the first void and lowest around noon (Fig. 1), which is probably due to differences in urinary volume during the different sampling times. Urinary creatinine is commonly used for normalising concentrations of biomonitoring results in urine spot samples, which would otherwise be greatly affected by varying diuresis^(31)^ and is a frequently used adjustment factor for urinary iodine when assessing iodine intake at a population level. However, urinary creatinine has been questioned as an adjustment factor alone when predicting iodine intake by measuring UIC^(2)^, since it varies depending on muscle mass, sex, ethnicity, age, and fluid and dietary protein intake^(32)^.

### Variability of daily iodine urinary excretion within and between individuals

It is already known that the variability of UIE within individuals is high^(9,15,33,34)^, which makes long-term individual iodine intake difficult to evaluate^(35)^. A Swiss study suggests that ten spot urine samples are needed in women for estimating 24UIE with 20 % precision^(33)^. They also calculated within-individual CV and found that it was comparable between e24UIE and 24UIE but higher for UIC^(33)^. Andersen *et al*.^(34)^ have concluded that at least seven urine samples (collected between 09.00 and 12.00 hours) are needed to estimate 24UIE individually in men with a precision range of ±20 %. Evaluations of iodine intake based on urinary iodine should therefore be used as an indicator on population level and not on an individual level. To evaluate individual iodine intake, self-reported food surveys may be used. A diet that completely excludes common iodine sources can probably predict a risk of iodine deficiency since a diet where you avoid fish and seafood, iodine-fortified salt and/or dairy products in Scandinavia increases the risk of iodine deficiency^(36)^. Thyroglobulin is a sensitive marker for iodine status and iodine intake at a group level, which follows a U-shaped relationship^(24)^, but it has limitations. As an individual marker, thyroglobulin has low sensitivity and specificity^(37)^.

### Strengths and limitations of the study

A limitation of the study is that our results reflect the food habits and iodine intake of the normal Swedish population, and this should be considered when interpreting the results. We excluded the data for one sampling day from an individual with extremely high iodine excretion on that day. This had only a minor impact on geometric mean and median values reported here. As would be expected, inclusion of this sampling day had a stronger impact on variability, and therefore we report ICC and CV both with and without this outlier sampling day. UIC levels in this study agree with other studies for the Swedish normal population^(26–29)^. Also, this study includes 24-h sampling from two different days per individual. To the best of our knowledge, this study is unique since it also measured the iHr over the day and calculated the ICC based on variability within and between individuals.

### Conclusions

According to our results, e24UIE is almost equal to 24UIE, which is considered the gold standard in this field. However, when using UIC from first void urine, e24UIE significantly underestimates 24UIE even though the correlation remains high. The iHr varies during the day and is lowest in the morning (first void) and highest after lunch (14.30 hours). Five urine samples per individual are needed to achieve 70–80 % of the true slope in an epidemiological study examining an outcome as function of iodine exposure using an individual-based design.
